# Editorial for the Special Issue: Colistin Resistance—The Need for a One Health Approach

**DOI:** 10.3390/antibiotics11091167

**Published:** 2022-08-30

**Authors:** Fabrizio Bertelloni, Barbara Turchi

**Affiliations:** Department of Veterinary Science, University of Pisa, 56124 Pisa, Italy

Colistin is an “old” antimicrobial belonging to the class of polymyxins, initially discovered in 1947. It was used in human medicine from the 1950s, but by the mid-1970s its use largely decreased due to its adverse effects and the availability of new and more secure drugs [1]. At the end of the last century, colistin was re-introduced for human treatment as consequence of the increase and spread of multidrug-resistant Gram-negative bacteria. Indeed, resistance to colistin was rare and linked to chromosomal mutations; it was not horizontally transmissible among bacteria [2].

Colistin was largely used in veterinary medicine for decades. It was used to treat gastrointestinal infections, particularly in farm animals within intensive husbandry systems. Furthermore, colistin was used as growth promoter; recently, this practice has been banned in many countries, but it is still adopted in some parts of the world [3].

In China in 2015, an *Escherichia coli* strain was isolated carrying a plasmid gene (*mcr-1*) responsible for colistin resistance [4]. Subsequently, numerous *mcr* homologous genes (*mcr-1* to *mcr-10*) and several variants were detected [5,6,7]. These genes were found all over the world in bacteria collected from human, animal, and environmental samples [5,6]. *E. coli* was determined to be the principal carrier of *mcr* genes, but they were also detected in other Gram-negative bacteria, such as *Klebsiella* spp. and *Salmonella* spp. [6]. Interestingly, retrospective investigations showed the circulation of *mcr* genes from the 1980s [5].

This Special Issue aimed to collect different works characterized by a One Health approach to better understand this emerging problem.

Ten articles were included in the Special Issue. Five manuscripts explored this topic in bacteria isolated from animals or animal products. Two articles reported on colistin resistance in human isolates. One article performed an investigation of IncI2 plasmids, which are most often involved in the carrying of *mcr*. One manuscript performed a comparison among different methods for the phenotypic detection of colistin resistance. Finally, a systemic review was included, exploring the problem of colistin dosage in human therapy.

An investigation of 174 *Salmonella*, belonging to eighteen serotypes and isolated from poultry farms in Serbia, was performed by Jovčić and colleagues. Only seven *S.* ser. Infantis strains were colistin resistant. No *mcr* genes were detected and no mutation events in the *phoPQ*, *pmrA*, and *mgrB* genes (known to confer colistin resistance) were observed. The molecular investigations revealed that four isolates, all belonging to the ST32 group, had mutations in the *pmrB* gene; the authors supposed that this could be a possible cause of phenotypic resistance to colistin, but the literature about this aspect is scant and it must be confirmed by further investigation. The authors evaluated the correlation of the expression levels of the *phoP*, *phoQ*, *mgrB*, *pmrA*, and *pmrB* genes as well as the colistin resistance; however, unclear and unambiguous results were obtained. The phenotypic colistin resistance identified was not fully supported by genetic analyses [8].

The contribution of Kalová and coworkers reports the characterization of 17 colistin-resistant bacteria from aquaculture products imported from Asia to the Czech Republic. Fourteen strains of *E. coli*, one *Klebsiella pneumoniae*, one *Acinetobacter baumannii,* and one *Acinetobacter nosocomialis* strain were tested. All isolates were positive for *mcr* genes; in particular, all *E. coli* carried *mcr-1.1*, and in one strain *mcr-3.5* was detected, too. *K. pneumoniae* was positive for *mcr-1.1* and *mcr-8.2*, whereas *A. baumannii* and *A. nosocomialis* harbored *mcr-4-3*. All but two of these bacteria carried the *mcr* genes on plasmids; the authors reported the involvement of different types of plasmids in the carriage of mobile colistin-resistance genes. This study highlights the risks of spreading antimicrobial-resistant bacteria that are associated with the import/export of food products [9].

The work of Macori and collaborators reports on the colistin resistance among *E. coli* isolated from bovine affected by diarrhea or mastitis. The authors performed whole-genome sequencing on 143 clinical isolates and showed no correlation between the genotypes and the clinical origin. Four isolates were positive for *mcr-1* gene, and all but one were phenotypically resistant to colistin too. The *mcr* genes were located on IncHI2 plasmids in all strains. In addition, these *E. coli* strains possessed several other resistance genes. These strains were isolated in France between 2004 and 2010 and these data provide one of the earliest cases of *mcr* circulation in Europe [10].

The study of Rodríguez-Santiago and coauthors reports the occurrence of colistin-resistant and *mcr-1* positive *E. coli* in pig stool from Mexico. From a collection of 175 ESBL *E. coli*, all collected from the same farm, 4 colistin-resistant strains were identified. All the strains carried the *mcr-1* genes on a conjugative 170-kb IncHI-2 plasmid co-carrying ESBL genes. The isolates belonged to three different serotypes and phylogroups. All the strains were phenotypically resistant to multiple antimicrobials and carried several resistance and virulence genes [11].

Bertelloni and colleagues performed a retrospective investigation of *Salmonella* strains collected from 2000 to 2020. Two hundred and thirty-six isolates were included, belonging to the subspecies *enterica*, *salamae*, *diarizonae*, and *houtenae*; the salmonellae originated from samples of birds, mammals, and reptiles collected from domestic animals, wildlife, and the environment. An overall prevalence of 17.79% colistin-resistant strains was detected, with no difference in relation to the years of isolation, subspecies, or origin of the samples. The *mcr* genes (*mcr-1*, *mcr-2*, *mcr-4*, *mcr-6*, and *mcr-8*) were detected in 5.93% of strains, but only 2.54% of the isolates were *mcr*-positive and colistin-resistant [12].

Tkadlec and coauthors performed a survey on the intestinal carriage of colistin-resistant Enterobacteriaceae in humans. The authors tested 1922 fecal samples of hospitalized patients collected between June 2018 and September 2019 in a tertiary care hospital in Czech Republic. The prevalence of colistin-resistant Enterobacteriaceae was 6.82%. The *mcr-1* gene was detected in only three colistin-resistant isolates and one colistin-susceptible *E. coli* isolate (an overall prevalence of 0.21%); the genes *mcr-2* to *mcr-8* were not detected. The four *E. coli* belonged to different sequence types, serotypes and phylogroups; all carried multiple plasmids and were phenotypically and genotypically resistant to multiple antimicrobials. The manuscript highlights the low circulation of colistin-resistant and *mcr*-positive Enterobacteriaceae in humans [13].

The work of Nogbou and colleagues reports the characterization of a colistin-resistant *Acinetobacter baumannii* isolated from a pediatric patient recovered in a tertiary hospital in Pretoria, South Africa. The strains were resistant to all the tested antimicrobials in addition to colistin. Whole-genome sequencing showed the presence of several antimicrobial-resistance genes. Interestingly, *mcr* genes were not found and no known colistin-associated resistance mutations were detected in the *lpx* or *pmr* genes. In this case, the colistin resistance was probably linked to a mutation in the *lpsB* gene, which was revealed by a whole-genome sequence analysis. Furthermore, the authors revealed an overexpression of the AdeABC efflux pump that could contribute to polymyxin resistance [14].

The work of Ricker and collaborators involves a global analysis of the IncI2 plasmid, which is more often involved in *mcr-1* transport. The sequences of 261 IncI2 plasmids, 121 harboring *mcr-1*, were extracted from available databases and examined. The data showed the circulation of two clusters of plasmids, one carrying resistance genes and the other one not. Most of the examined plasmids belonging to the first cluster had *mcr* genes; other resistance genes were present but less frequently. Some of these IncI2 plasmids seemed to possess a particular sequence that may favor the acquisition of *mcr* genes [15].

The evaluation of four different methods for colistin susceptibility tests was performed by García-Meniño and colleagues. The four employed methods were: a disc-diffusion test (with two different breakpoints), the UMIC, an E-test, and MicroScan. The broth microdilution method (BMD) was used as the reference method, and 75 susceptible and 75 *mcr*-positive *E. coli* were tested. The disc-diffusion test yielded a high number of false susceptible strains, applying the standard cut-off value of ≤11 mm. MicroScan, the E-test, and the UMIC showed good results, with a good correlation with the BMD. The reference method was unable to detect some *mcr*-positive strains and the authors suggested lowering the cut-off value to >1 mg/L to increase the detection rate of *mcr*-carrying *E. coli* [16].

Although colistin was reintroduced for human therapy as a last-resort antimicrobial against multidrug-resistant-Gram-negative infections, a limited number of recent articles have been published regarding the dosage regimen of this molecule in humans. Haseeb and coauthors proposed a systemic review on this specific topic. They included nineteen articles, with only one proposing a dosing schedule for pediatric populations, published between 2005 and 2021. Among them, 16 were cohort studies, 1 was an RCT, and 2 were case reports. The authors concluded that a loading dose of 9 million international units (MIU) of colistin followed by a maintenance dose of 4.5 MIU every 12 h could be considered the most appropriate dosing strategy [17].

This Special Issue collects multidisciplinary investigations on colistin resistance, on the rise and spread of the new *mcr* genes, and on other mechanisms that might be involved in a phenotypic colistin resistance. Considering the One Health concept, an integrated approach is essential to face this emerging problem, due to the increasing amount of general antimicrobial resistance.
